# Transduodenal Sphincteroplasty and Transampullary Septectomy for Papillary Stenosis

**DOI:** 10.1155/1996/89190

**Published:** 1996

**Authors:** S. B. Kelly, B. J. Rowlands

**Affiliations:** Department of Surgery Institute of Clinical Science Royal Victoria Hospital Grosvenor Road Belfast BT12 6BJ Ireland

## Abstract

Twenty patients received transduodenal sphincteroplasty and transampullary septectomy between 1987 and 1993. Seven patients had post-cholecystectomy pain which was much improved or abolished in 5 of 7 patients at a mean follow-up of 4 years and 5 months. Four of five patients with chronic pancreatitis were improved at 3 years and 2 months. Three of five patients with recurrent acute pancreatitis were improved at 4 years and 5 months. One of three patients with chronic abdominal pain of hepatobiliary origin was improved at 3 years. Transduodenal sphincteroplasty and transampullary septectomy can relieve pain in patients with post-cholecystectomy pain, recurrent acute pancreatitis, chronic pancreatitis, and chronic abdominal pain of hepatobiliary origin, presumably by improving drainage of the obstructed ducts.


*HPB Surgery*, 1996, Vol.9, pp.199–207
Reprints available directly from the publisher
Photocopying permitted by license only

© 1996 OPA (Overseas Publishers Association)
Amsterdam B.V. Published in The Netherlands
by Harwood Academic Publishers GmbH
Printed in Malaysia

### Transduodenal Sphincteroplasty and Transampullary Septectomy for Papillary Stenosis

S.B. KELLY and B.J. ROWLANDS

Department of Surgery, Institute of Clinical Science, Royal Victoria Hospital,
Grosvenor Road, Belfast. BT12 6BJ

*(Received 10 February 1994)*

Twenty patients received transduodenal sphincteroplasty and transampullary septectomy between 1987 and 1993. Seven patients had post-cholecystectomy pain which was much improved or abolished in 5 of 7 patients at a mean follow-up of 4 years and 5 months. Four of five patients with chronic pancreatitis were improved at 3 years and 2 months. Three of five patients with recurrent acute pancreatitis were improved at 4 years and 5 months. One of three patients with chronic abdominal pain of hepatobiliary origin was improved at 3 years. Transduodenal sphincteroplasty and transampullary septectomy can relieve pain in patients with post-cholecystectomy pain, recurrent acute pancreatitis, chronic pancreatitis, and chronic abdominal pain of hepatobiliary origin, presumably by improving drainage of the obstructed ducts.

KEY WORDS: Transduodenal sphincteroplasty transampullary septectomy papillary stenosis

### INTRODUCTION

The indications for performing transduodenal sphincteroplasty and transampullary septectomy are controversial. What is known, however, is that a carefully performed ablation of the biliary and pancreatic components of the papilla and its sphincter will lead to relief of episodic, incapacitating upper abdominal pain in selected patients[1]. Langenbuch[2] first suggested that stenosis of the sphincter of Oddi might cause biliary-like pain. Ever since, this condition has been recognised as a cause of upper abdominal pain occurring either as an isolated abnormality, or in association with gallbladder disease, pancreatitis, or both[3].

Obstruction to the terminal bile duct causing pain with or without jaundice can be treated by endoscopic papillotomy[4]. On the other hand, obstruction to the terminal pancreatic duct causing pain with or without hyperamylassaemia requires a transduodenal operation. Transduodenal sphincteroplasty and transampullary septectomy should permanently abolish stenosis at both sites[5].

The aim of this study was to assess the effectiveness of transduodenal sphincteroplasty and transampullary septectomy in patients with post-cholecystectomy pain, recurrent acute pancreatitis, chronic pancreatitis and chronic abdominal pain of hepatobiliary origin. This series is unusual in that the diagnosis was made by percutaneous transhepatic cholangiogram (PTC) in half of the patients.

### PATIENTS AND METHODS

Twenty patients received transduodenal sphincteroplasty and transampullary septectomy between 1987 and 1993. All patients had severe pain for more than two years which was refractory to medical therapy. Mean age was 44 years (range 20–62 years) and sex distribution was 10M:10F. Operation was indicated

*Correspondence to*: Professor B.J. Rowlands, Department of Surgery, Institute of Clinical Science, Royal Victoria Hospital, Grosvenor Road, Belfast. BT12 6BJ.

for one of four different syndromes (Table 1). The patients were followed up at the out patient department and by telephone.

**Table 1** Indications for pancreatic sphincteroplasty.

| | *Patients* | *M* | *F* | *Mean age (yrs)* |
|---|---|---|---|---|
| Post-cholecystectomy pain | 7 | 3 | 4 | 43 |
| Recurrent acute pancreatitis | 5 | 3 | 2 | 45 |
| Chronic pancreatitis | 5 | 3 | 2 | 40 |
| Papillary Stenosis diagnosed at cholecystectomy | 1 | 0 | 1 | 58 |
| Papillary stenosis diagnosed before cholecystectomy | 2 | 0 | 2 | 45 |

*1) Post-cholecystectomy pain*

Seven patients had chronic disabling pain in the epigastrium or right hypochondrium which radiated through to the back and was accompanied by nausea and vomiting. These patients were followed up for a mean of 4 years and 7 months (range 1 year and 2 months–6 years and 8 months) after sphincteroplasty. Nifedipine was successful at relieving the pain in only one of three patients. Intermittent jaundice was present in three patients. All patients had a cholecystectomy performed for gallstones 8 months to 13 years earlier. Only one patient had an exploration of the common bile duct. Cannulation of the bile duct at ERCP in two patients produced pain identical to that of which they had been complaining. One of these patients had an endoscopic sphincterotomy, but although this helped initially, it was only effective for 6 weeks and therefore a sphincteroplasty was performed with an excellent result. Liver biopsy was performed in two patients and was normal. Other investigations included liver function tests, serum amylase, gastroscopy, barium meal, barium enema, and oesophageal manometry.

*2) Recurrent acute pancreatitis*

Five patients had recurrent acute pancreatitis during the previous 3-14 years. These patients were followed up for a mean of 3 years and 4 months (range 9 months–6 years) after sphincteroplasty. Attacks of pancreatitis were usually accompanied by hyperamylassaemia and between episodes patients were largely free of pain. None were noted to have pancreatic exocrine or endocrine insufficiency. One patient had several episodes of jaundice. Two patients had pancreas divisum on ERCP but there was no evidence of chronic pancreatitis. Two patients developed pseudocysts and both had a cyst-gastrostomy performed. One patient also had a pancreatic necrosectomy. The aetiology of the pancreatitis was due to gallstones (2), pancreas divisum (2), and alcohol (1). Other investigations included liver function tests, serum amylase, gastroscopy, barium meal, barium enema, small bowel series, and PABA test.

*3) Chronic pancreatitis*

Five patients had upper abdominal pain radiating through to the back, accompanied by nausea and vomiting for 8 months to 17 years. These patients were followed up for a mean of 2 years and 7 months (range 1 year and 4 months–5 years and 11 months). Intermittent exacerbations of pain were generally not accompanied by an elevation of the serum amylase. Three patients had evidence of pancreatic exocrine insufficiency and two patients also had endocrine insufficiency. Two patients had previously undergone distal pancreatectomy and splenectomy and one patient had drainage of several pancreatic abscesses. The aetiology of the pancreatitis was due to alcohol (3), idiopathic (1), and pancreas divisum (1). The patient with pancreas divisum developed a pancreatico-pleural fistula. ERCP revealed a dilated, ectatic pancreatic duct with a pseudocyst at the tail of the pancreas draining into the fistula (Fig. 1). One patient had a small pseudocyst in the tail of the pancreas and a large splenic cyst (Fig. 2). Other investigations included liver function tests, serum amylase, gastroscopy, PABA test, and in one patient a percutaneous ultrasound-guided pancreatogram was attempted but this was unsuccessful.

*4) Chronic abdominal pain of hepatobiliary origin*

Three patients with chronic abdominal pain of hepatobiliary origin were followed up for a mean of 3 years and 3 months (range 1 year and 6 months–6 years and 4 months). One patient had a cholecystectomy for gallbladder stones. Operative cholangiogram showed normal anatomy with no stones but no passage of contrast into the duodenum. Upon inspection at laparotomy, excessive fibrosis of the papilla was found and so a sphincteroplasty was performed. Stones were present in the gallbladder. Two patients presented with upper abdominal pain, vomiting and cholestatic liver function tests. Pre-operative PTC confirmed papillary stenosis (Fig. 3). Both patients had gallbaldder stones.

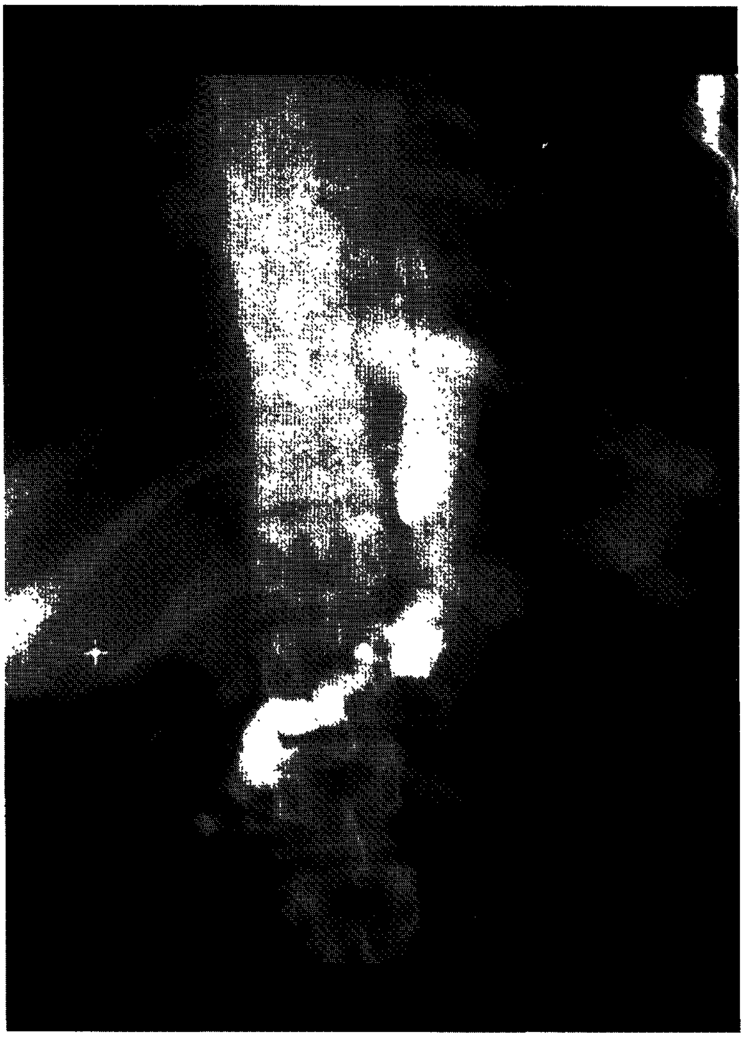

**Figure 1** ERCP demonstrating pancreas divisum, chronic pancreatitis and a pseudocyst at the tail of the pancreas connecting with a pancreatico-pleural fistula.

*Diagnosis*

The various tests which were used for diagnosis, either singly or in combination, are listed in Table 2. The Nardi test[6] (morphine-prostigmine provocation test), which is somewhat controversial, relies on the reproduction of pain and a sharp rise in serum pancreatic enzymes (amylase, lipase or amidase). A positive test requires a relatively normal pancreas secreting against a closed and competent sphincter. Serum amylase was measured and symptoms recorded for 1 hour after an intramuscular injection of saline and for 4 hours after an intramuscular injection of morphine 10 mg plus prostigmine 1 mg. The test was considered positive if the morphine-prostigmine injection reproduced pain and caused a fourfold elevation in serum amylase.

*Operative technique*

An oblique incision is made in the second part of the duodenum and the papilla is identified on the medial wall. A biliary sphincteroplasty 15-20 mm in length is performed and the mucosae of the duodenum and common bile duct are coapted with 5/0 Vicryl sutures. The orifice of the duct of Wirsung is then identified on the lower lip of the ampulla at 5 o'clock. The common septum between the terminal portions of the bile duct and pancreatic duct is then incised for 10–15 mm, using fine-pointed scissors and sutures of 5/0 Vicryl to coapt the mucosae. At the end of the procedure both ducts should be widely patent, presenting a double-barrelled appearance (Figure 4). The duodenotomy is closed in two layers with 2/0 Vicryl.

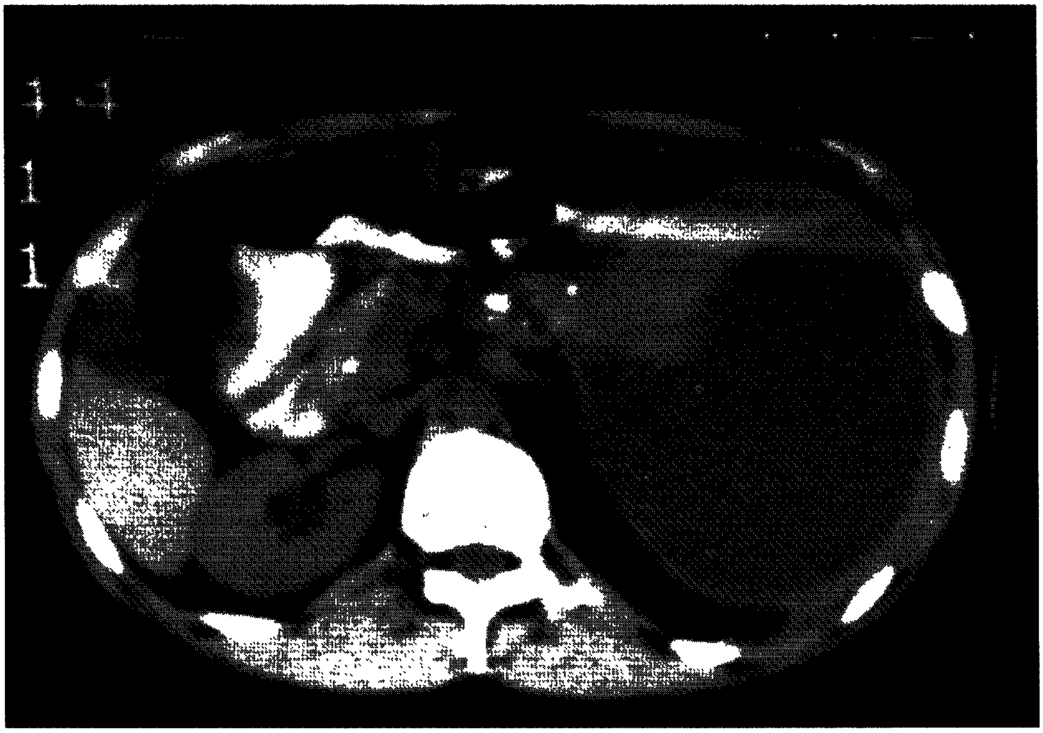

**Figure 2** CAT scan showing a small pancreas containing flecks of calcium, a dilated pancreatic duct, a small pseudocyst in the tail of the pancreas, and a large splenic cyst.

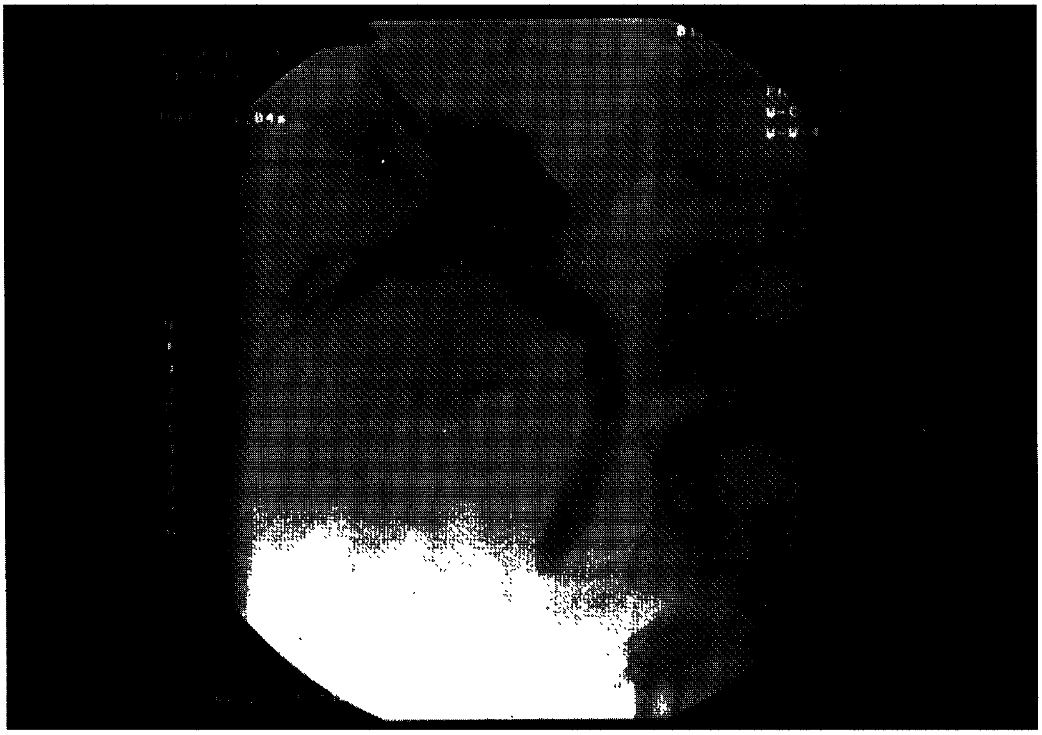

**Figure 3** Percutaneous transhepatic cholangiogram showing delayed emptying at the lower end of the common bile duct due to papillary dysfunction.

**Table 2** Method of diagnosis.

| *Method* | *n* |
|---|---|
| USS | 15 |
| CT scan | 7 |
| Oral cholecystogram | 1 |
| PTC | 10 |
| ERCP | 14 |
| PTC + ERCP | 6 |
| Intravenous cholangiogram | 2 |
| Operative cholangiogram | 4 |
| Nardi test | 1 |
| HIDA scan | 2 |
| Inspection at laparotomy | 1 |

**Table 3** Operations performed.

| | *n* | *Sphincteroplasty* | | *Cholecys-* |
|---|---|---|---|---|
| | | *Major* | *Minor* | *tectomy* |
| Post-cholecystectomy pain | 7 | 7 | 0 | 0 |
| Recurrent acute pan-creatitis | 5 | 5 | 2 | 5 |
| Chronic pancreatitis | 5 | 4 | 1 | 4 |
| Chronic abdominal pain | 3 | 3 | 0 | 3 |

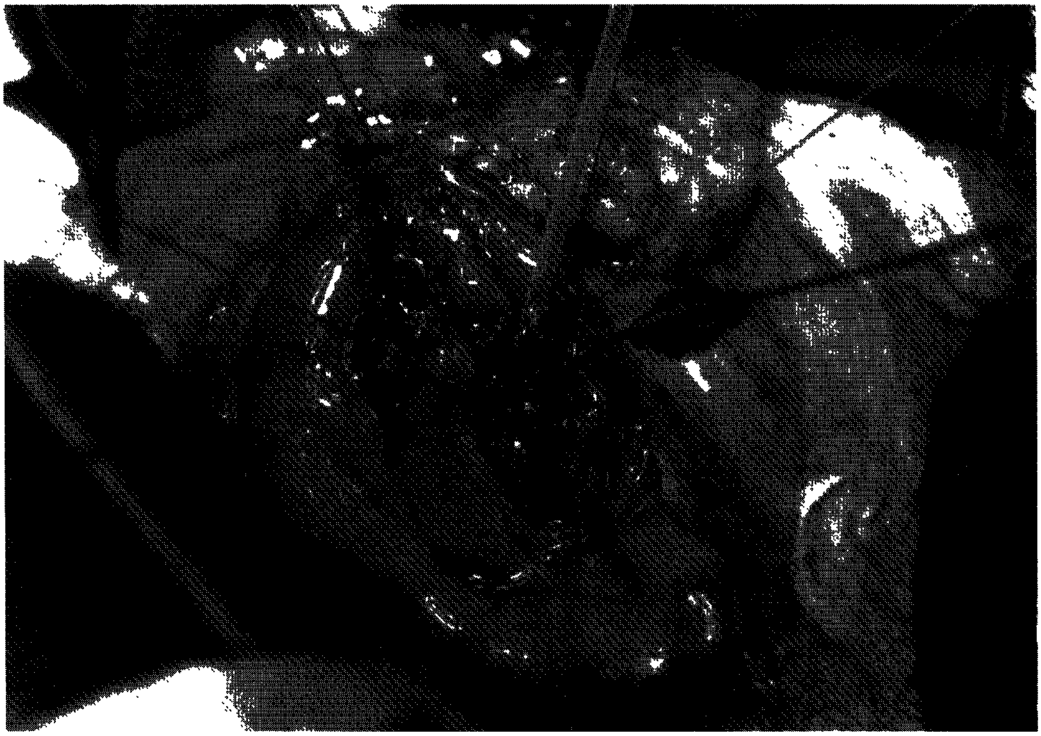

**Figure 4** Operative photograph showing completed transduodenal sphincteroplasty and transampullary septectomy with a catheter in the common bile duct and a lacrimal probe in the pancreatic duct.

The following technical points have been found helpful. If the papilla is difficult to find, the supraduodenal bile duct is opened and an 8 French gauge Jaques catheter is passed from above. This manoeuvre also helps to identify a stenosed bile duct orifice if the initial sphincteroplasty enters the pancreatic duct. If either the major or minor pancreatic duct is difficult to find, it may be located after i.v. administration of secretin 1 U/kg, which produces a brisk flow of pancreatic juice within one minute. If the orifice of the pancreatic duct is stenosed, it may require the passage of fine lacrimal probes before it will accept a cannula.

### RESULTS

There were no deaths in this series. Three patients developed hyperamylassaemia post-operatively and one patient developed a pancreatic pseudocyst which required a cyst-gastrostomy. Another patient developed pancreatitis with abscess formation and obstruction to the second part of the duodenum. She settled on conservative treatment with nasogastric suction and antibiotics.

The mean hospital stay was 13 days (range 8–45 days). The operations performed are given in Table 3

**Table 4** Outcome of pancreatic sphincteroplasty.

| | *n* | *Mean follow-up (Yrs, mths)* | *Improved* | *Recurred* |
|---|---|---|---|---|
| Post-cholectstectomy pain | 7 | 4,5 | Ex4, Go1 | 2 |
| Recurrent acute pancreatitis | 5 | 4,5 | Ex3 | 2 |
| Chronic pancreatitis | 5 | 3,2 | Ex3,Go1 | 1 |
| Chronic abdominal pain | 3 | 3,0 | Ex1 | 2 |

Ex = Excellent (complete resolution of symptoms)
Go = Good (significant improvement in symptoms)

and the results in Table 4. Nineteen patients had a sphincteroplasty of the major papilla and three patients had a sphincteroplasty of the minor papilla. Twelve patients also had a cholecystectomy. In one patient, the orifice of the common bile duct couldn't be identified and so a probe was passed via a supraduodenal choledochotomy.

### *1) Post-cholecystectomy pain*

Five of the seven patients with post-cholecystectomy pain were improved by their operation (Table 4). Four had an excellent result (complete relief of symptoms) and one had a good result (marked improvement in symptoms). One patient continued to have upper abdominal pain and vomiting postoperatively and had an ERCP performed nine months later. This showed a normal pancreatic duct a dilated common bile duct with delayed emptying and narrowing at its lower end. An endoscopic sphincterotomy was performed which improved drainage, however, five months later, she continued to have pain and cholangitis. A repeat ERCP showed that the previous sphincterotomy was widely patent but that the common bile duct was slow to empty. Another patient continued to have pain and he had good pain relief following a coeliac plexus block.

### *2) Recurrent acute pancreatitis*

Of the five patients who had operations for recurrent acute pancreatitis, three had complete relief of their symptoms and two had recurrent symptoms. Two were found to have pancreas divisum and both had sphincteroplasties of the major and minor papillae. Both patients had recurrent symptoms following operation. There was no evidence of chronic pancreatitis on ERCP in either patient. One of these patients had a strongly positive Nardi test. Another patient developed a pseudocyst 4 months post-operatively and had a cyst-gastrostomy performed. One patient with recurrent acute pancreatitis and excessive alcohol intake stopped drinking five years before sphincteroplasty. However, despite this, his pain was not relieved by sphincteroplasty.

### *3) Chronic pancreatitis*

Four of five patients with chronic pancreatitis were improved following operation and one had recurrent symptoms. A 20-year-old female with pancreas divisum was found to have a fistulous tract extending from the superior aspect of the body of the pancreas, posterior to the splenic artery, and extending up behind the diaphragm into the chest. The pancreas was transected at its neck and the pancreatic duct was found to be dilated. A spleen-preserving distal pancreatectomy was performed and the fistula was identified and resected. An attempt was made to pass a catheter into the duodenum via the pancreatic duct, however, this was unsuccessful and so the duodenum was opened and a sphincteroplasty of the minor papilla was performed. Finally, a Roux-en-Y pancreatico-jejunostomy was performed. A 24-year-old male had a large splenic cyst and underwent a splenectomy and distal pancreatectomy with removal of the splenic cyst. In addition, he had a Roux-en-Y pancreatico-jejunostomy, removal of pancreatic duct calculi and a transduodenal sphincteroplasty and transampullary septectomy. The patient with recurrent symptoms had a previous history of excessive alcohol intake and had multiple pancreatic cysts, abscesses and fistulae, following which he had undergone a distal pancreatectomy. He stopped drinking 5 years before sphincteroplasty. Two other patients with chronic pancreatitis and excessive alcohol intake stopped drinking 8 months and 8 years respectively prior to sphincteroplasty. Both patients had a successful result following sphincteroplasty.

### *4) Chronic abdominal pain of hepatobiliary origin*

One of three patients with chronic abdominal pain of hepatobiliary origin had an excellent response following sphincteroplasty and two patients had recurrent symptoms.

### DISCUSSION

Surgical procedures on the sphincter of Oddi and the ampulla of Vater have had a high failure rate since there are few specific objective criteria or diagnostic tests for selecting surgical candidates[7]. In this series, the decision to recommend surgery was based on a careful history, physical examination, and laboratory evaluation, including liver function tests and pancreatic enzymes. Selection criteria were based primarily on clinical symptoms such as severe, episodic, incapacitating upper abdominal pain after a cholecystectomy, or recurrent episodes of unexplained hyperamylassaemia with pain. All patients had severe pain for greater than two years which was refractory to medical therapy.

Important diagnostic tests included ERCP and PTC which demonstrated either: 1) an abnormality of the papilla (e.g. papillary stenosis or pancreas divisum), or 2) dilatation with delayed emptying of the contents of the biliary or pancreatic duct, or 3) reproduction of symptoms at the time of ERCP. An interesting feature of this series is that the diagnosis was made by PTC in half of the patients (Table 2). In most other series, the diagnosis was usually made by ERCP and we are not aware of other studies which have used PTC for diagnosis.

ERCP manometry can predict which patients will respond to treatment, either surgical or endoscopic. Geenen[8] performed a randomised prospective study of 47 patients who had endoscopic sphincterotomy performed for sphincter of Oddi dysfunction following cholecystectomy. He showed that in patients with increased basal sphincter pressure, sphincter dysfunction was a major cause of symptoms, because the symptoms improved substantially or disappeared after sphincterotomy. Roberts-Thompson[9] performed endoscopic sphincterotomy in 45 patients with sphincter of Oddi dysfunction following cholecystectomy, with a success rate of 50%. Carr-Locke[10] treated 30 patients with the same condition with a success rate of 18/30 (60%).

The pathogenesis of upper abdominal pain following cholecystectomy, the so-called post-cholecystectomy syndrome, remains obscure[5]. It has been suggested that the pain is pancreatic in origin and that the chronic passage of gallstones might lead to inflammation and fibrosis of the transampullary septum within the papilla of Vater and critical stenosis of the main pancreatic duct of Wirsung.

The clearest indication for pancreatic sphincteroplasty is probably recurrent idiopathic pancreatitis in patients with suspected papillary stenosis or pancreas divisum[11]. ERCP is a crucial investigation in such patients, and the inability of an experienced endoscopist to cannulate the pancreatic duct may be taken as evidence of stenosis[12]. Cannulation of the accessory papilla was successful in all three patients with pancreas divisum in this series. The reason for the association between recurrent pancreatitis and pancreas divisum is not known. It has been suggested that inadequate drainage of the dorsal pancreas through the dorsal pancreatic duct and a relatively stenosed minor papilla could cause pancreatitis[13,14]. Therefore, it has been proposed that enlargement of the outflow tract of the duct of Santorini by sphincteroplasty might benefit patients with recurrent pancreatitis due to pancreas divisum[15,16]. Sphincteroplasty of the minor papilla should be sufficient if the pancreatitis is caused by poor drainage through a relatively stenosed minor papilla. However, in most series, including our own, a sphincteroplasty of the major papilla and duct of Wirsung has been added for completeness[13,16]. In Warshaw's experience, sphincteroplasty of the accessory ampulla was successful in treating 18 of 19 patients with pancreas divisum and clearcut stenosis at the minor papilla[17]. In our series, however, only 1 of 3 patients with pancreas divisum had a successful outcome following accessory sphincteroplasty. On the basis of clinical presentation, it is impossible to separate patients with recurrent pancreatitis into Sarles' type II or III. Yet, this is of the utmost importance, since the former, with acute recurrent disease and a normal parenchyma, should respond well to a decompressive procedure when obstruction is present, whereas the latter, with progressive pathology, will eventually have recurrent symptoms[18].

The finding that diarrhoea and previous surgery could singly, or in combination, result in a significantly poorer outcome may signal that these may be patients with early exocrine insufficiency and longstanding disease suggestive of Sarles' type III. The poor results associated with alcoholism and drug dependency are well recognised, and, again, should alert us to the fact that we are dealing with a type of disease which will not respond to decompressive measures[18].

In established chronic pancreatitis, sphincteroplasty has a very limited role. It can facilitate retrograde pancreatography (if ERCP is inadequate) and extraction of ductal calculi and should relieve a localised ductal stenosis immediately adjacent to the papilla. Long-term relief of symptoms may be anticipated in about 40% of patients with relatively mild disease, especially if they abstain from alcohol, and the short-term benefit is greater[11,19]. In the present series, four of five patients have had a useful response. Sphincteroplasty was only an adjunct to other pancreatobiliary procedures in two patients.

Relief of symptoms after endoscopic sphincterotomy has been reported in patients with recurrent acute pancreatitis[20]. Balloon dilatation of the sphincter has been attempted, but this is not effective and is associated with a high frequency of complications, especially pancreatitis. Stent placement across the papilla has been shown to work in 5 of 12 patients[20]. Endoscopic treatment of chronic pancreatitis is aimed at alleviating outflow obstruction caused by ductal strictures, stones, or papillary stenosis. Endoscopic sphincterotomy of the pancreatic duct sphincter precedes dilatation using either dilating catheters or balloons and stent placement. In addition, self-expandable metallic wallstents have recently been used. Stones may be extracted or fragmented by using extracorporeal shock wave lithotripsy (ESWL).

The morbidity from transduodenal sphincteroplasty and transampullary septectomy remains high, even in the hands of those who have mastered the technique[21]. Potential complications include: 1) acute pancreatitis, 2) transient postoperative hyperamylassaemia, 3) an infected collection of pancreatic juice, and 4) duodenal fistula. The absence of operative mortality in this series and the relatively low incidence of serious morbidity (10%) are consistent with meticulous attention to details of operative and perioperative care. Others have commented on the relatively high incidence of pancreatitis associated with procedures on the papilla of Vater[22]. The low incidence of postoperative pancreatitis in this series (10%) was probably due to gentle handling of the papilla and identification and enlargement of the opening of the duct of Wirsung or Santorini. In addition, operative pancreatography was only used in one patient[23].

The Nardi test[6] has been found to be of use in selecting patients with post-cholecystectomy pain for sphincteroplasty[11,24-26]. We only used this test in one patient with recurrent acute pancreatitis. The test was positive pre-operatively (reproduction of pain plus a fourfold elevation of serum amylase), however, sphincteroplasty was unsuccessful.

In conclusion, surgery may offer the best means of treating this difficult group of patients. However, there is a learning curve associated with this procedure, which requires a surgeon with a special interest and expertise in treating this condition. He must be prepared to follow these patients up and to re-operate if necessary. If this expertise is lacking, then endoscopic sphincterotomy may be offered as an alternative treatment. The overall long-term clinical success rates achieved with therapeutic endoscopy are probably somewhat lower than those achieved by operative treatment. Moreover, endoscopic treatment may have to be repeated for recurrent symptoms. Nevertheless, endoscopic treatment seems to be reasonably safe and technically feasible, and provides relief in a substantial proportion of patients. In addition, it may be preferred by patients and is probably less expensive. ERCP manometry can predict which patients will respond to treatment, either surgical or endoscopic. However, this technique is not widely available, except in a few specialist centres.

### REFERENCES

1. Moody, F.G. (1990): The postcholecystectomy syndrome. In: Moody FG, ed. Surgical treatment of digestive disease. 2nd ed. Chicago: Year Book Medical Publishers. 298–309.
2. Langenbuch, C. (1884): Einiges uber operationem am gallensystem. *Klin Wochenschr.* **21**: 809–11.
3. Gregg, J.A., Clark G., Barr, C., McCartney, A., Milano, A. and Volejak, C. (1980): Post cholecystectomy syndrome and its association with ampullary stenosis. *Am J Surg.* **139**: 374–8.
4. Roberts-Thomson, I.C. and Toouli, J. (1985): Is endoscopic sphincterotomy for disabling biliary-type pain after cholecystectomy effective? *Gastrointest Endosc.* **31**: 370–3.
5. Moody, F.G., Becker, J.M. and Potts, J.R. (1983): Transduodenal sphincteroplasty and transampullary septectomy for postcholecystectomy pain. *Ann Surg.* **197**: 627–36.
6. Nardi, G.L. and Acosta, J.M. (1966): Papillitis as a cause of pancreatitis and abdominal pain: role of evocative test, operative pancreatography and histologic evaluation. *Ann Surg.* **164**: 611–21.
7. Nussbaum, M.S., Warner, B.W., Sax, H.C. and Fischer, J.E. (1989): Transduodenal sphincteroplasty and transampullary septotomy for primary sphincter of Oddi dysfunction. *Am J Surg.* **157**: 38–43.
8. Geenen, J.E., Hogan, W.J., Dodds, W.J., Toouli, J. and Venn, R.P. (1989): The efficacy of endoscopic sphincterotomy after cholecystectomy in patients with sphincter of Oddi dysfunction. *N Engl J Med.* **320**: 82–7.
9. Roberts-Thompson I.C. (1984): Endoscopic sphincterotomy of the papilla of Vater: an analysis of 300 cases. *Aust N Z J Med.* **14**: 611–7.
10. Carr-Locke D.L., Bailey I., Neoptolemos J., Lesse T. and Heath D. (1986): Outcome of endoscopic sphincterotomy for papillary stenosis. *Gut.* **27**: A 1280 (abstract).
11. Williamson, R.C.N. (1988): Pancreatic sphincteroplasty: indications and outcome. *Ann Roy Coll Surg Eng.* **70**: 205–11.
12. Gregg, J.A., Taddeo, A.E., Milano, A.F., McCartney, A.J., Santoro, B.T., Frager, S.H. and Capobianco, A.G. (1977): Duodenoscopy and endoscopic pancreatography in patients with positive morphine prostigmine tests. *Am J Surg.* **134**: 318–21.
13. Richter, J.M., Schapiro, R.H., Mulley, A.G. and Warshaw, A.L. (1981): Association of pancreas divisum and pancreatitis, and its treatment by sphincteroplasty of the accessory ampulla. *Gastroenterology.* **81**: 1104–10.
14. Cotton, P.B. (1980): Congenital anomaly of pancreas divisum as cause of obstructive pain and pancreatitis. *Gut.* **21**: 104–14.
15. Gregg, J.A. (1977): Pancreas divisum: its association with pancreatitis. *Am J Surg.* **134**: 539–43.
16. Gregg, J.A., Monaco, A.P. and McDermott, W.V. (1983): Pancreas divisum. Results of surgical intervention. *Am J Surg.* **145**: 488–92.

17. Warshaw, A.L., Richter, J.M. and Schapiro, R.H. (1983): the cause and treatment of pancreatitis associated with pancreas divisum. Ann Surg. 198: 443-52.
18. Nardi, G.L., Michelassi, F. and Zannini, P. (1983): Transduodenal sphincteroplasty. 5–25 year follow-up of 89 patients. *Ann Surg*. **198**: 453–61.
19. Bagley, F.H., Braasch, J.W., Taylor, R.H. and Warren, K.W. (1981): Sphincterotomy or sphincteroplasty in the treatment of pathologically mild chronic pancreatitis. *Am J Surg*. **141**: 418–22.
20. Geenen J.E. and Rolny P. (1991): Endoscopic therapy of acute and chronic pancreatitis. *Gastrointestinal Endoscopy*. **37**: 377–82.
21. Moody, F.G., Vecchio, R., Calabuig, R. and Runkel, N. (1991): Transduodenal sphincteroplasty with trans-ampullary septectomy for stenosing papillitis. *Am J Surg* **161**: 213–8.
22. Thomas, C.G. Jr., Nicholson, C.P. and Owen, J. (1971): Effectiveness of choledochoduodenostomy and transduodenal sphincterotomy in the treatment of benign obstruction of the common duct. *Ann Surg*. **173**: 845–856.
23. Cooper, M.J. and Williamson, R.C.N. (1983): The value of operative pancreatography. *Br J Surg*. **70**: 577–80.
24. Lo Giudice, J.A., Geenen, J.E., Hogan, W.J. and Dodds, W.J. (1979): Efficacy of the morphine-prostigmin test for evaluating patients with suspected papillary stenosis. *Dig Dis Sci*. **24**: 455–8.
25. Madura, J.A., McCammon, R.L., Paris, J.M. and Jesseph, J.E. (1981): The Nardi test and biliary manometry in the diagnosis of pancreaticobiliary sphincter dysfunction. *Surgery*. **90**: 588–95.
26. Steinberg, W.M., Salvato, R.F. and Toskes, P.P. (1980): The morphine-prostigmin provocation test - is it useful for making clinical decisions? *Gastroenterology*. **78**: 728–31.

